# Evaluation of availability, price, and affordability of cardiovascular, diabetes, and global medicines in Abuja, Nigeria

**DOI:** 10.1371/journal.pone.0255567

**Published:** 2021-08-12

**Authors:** Nkeiruka Grace Osuafor, Chinwe Victoria Ukwe, Mathew Okonta

**Affiliations:** Department of Clinical Pharmacy and Pharmacy Management, Faculty of Pharmaceutical Sciences, University of Nigeria, Nsukka, Nsukka, Nigeria; University of South Carolina, UNITED STATES

## Abstract

**Objective:**

To assess the availability, price, and affordability of cardiovascular, diabetes, and global medicines in Abuja, Nigeria.

**Methods:**

A cross-sectional survey involving 27 private pharmacies, 13 public pharmacies, and 25 private hospital pharmacies in Abuja was conducted using the standardized World Health Organization/Health Action International methodology. The availability percentage for each pharmacy sector and each medicine was analyzed. The median price ratio (MPR) (ratio of the median price to the international reference prices) of the medicines were evaluated accordingly. Affordability was assessed by calculating the number of days’ wages the lowest-paid unskilled government worker required to purchase a month worth of the standard treatment for a chronic condition.

**Results:**

The availability of cardiovascular (CV) medicines ranged from 28.4% (in private hospital pharmacies) to 59.9% (in private pharmacies). There was mixed variability in the mean availability of Originator Brands (OBs) and Lowest Priced Generics (LPGs) anti-diabetic drugs with the highest availability being OBs 36% and LPGs 40.2%, in private pharmacies and public pharmacies, respectively. The availability of global drugs ranged from 49.7% in private hospitals to 68.8% in private pharmacies. Two cardiovascular and four global medicines had greater than 80% availability across the pharmaceutical sectors. The median price ratio for OBs and LPGs was 9.60 and 1.72 for procurement, it was 8.08 and 2.60 in private pharmacies, 13.56 and 2.66 in public hospitals, and 16.38 and 7.89 in private hospitals. The percentage markup on LPG was 49.4% in public hospitals, 51.4% in private pharmacies, and 323% in private hospitals. Only nine medicines in both public hospitals and private pharmacies and two in the private hospital pharmacies required less than the daily wage of the lowest-paid government worker.

**Conclusion:**

The availability of cardiovascular, diabetes, and global medicines was below 80% across the different pharmaceutical sectors in Abuja and the medicines were unaffordable. Although the prices were generally exorbitant, private pharmacies offered the best options in terms of availability, pricing, and affordability of medicines. Therefore, the results of this study emphasize the pertinence of enforcing policies that facilitate the availability, pricing, and affordability of cardiovascular, diabetes, and global medicines.

## Introduction

Medicines play an indispensable role in the improvement of health, the preservation of lives, the enhancement of public welfare, the promotion of trust, and participation in healthcare services [1]. The availability, pricing, and affordability of medicines are particularly important in this regard as it significantly affects the survival rate of populations from diseases. In the political declaration on non-communicable diseases (NCDs) in 2011 [2], heads of government committed to ensuring equitable access to medicines for the management of NCDs. Similarly, the Global Action Plan recommends 80% access to medicines as the global target for the prevention and control of NCDs [3]. Access to medicines is an inextricable prerequisite for the achievement of other global health targets such as the 25% reduction in mortality rates due to NCDs [4], and the provision of drug therapy to efficiently manage cardiovascular diseases (CVDs) and diabetes [5].

Access to medicines encompasses the constant availability and affordability of essential medicines at either public or private health facilities, that are within a one-hour walking distance from the homes of the population [6]. Access to medicine constitutes a core component of the right to health [7]. Unfortunately, one-third of the world’s population does not have access to medicine, and 50% of this demographic live in low and middle-income countries [8]. For example, according to a recent study on access to medicine and affordable treatment for acute and chronic diseases in 36 developing and middle-income countries, the availability of generics in the public sector ranged from 29.4% in Africa to 54.4% in the Americas [9]. This poor availability and affordability of medicines in African countries can be attributed to several factors, including inadequate health financing systems, inefficient medicine supply systems, and out-of-pocket payment for drugs [10]. In Nigeria, the total healthcare expenditure as a percentage of gross domestic product (GDP) in 2013 was 3.7% [11], and the majority of the drugs used in the country are imported [12]. A recent study on cardiovascular and diabetes drug use in Abuja reported 92.8% out-of-pocket payment from the 1008 prescriptions studied [13].

The increasing prevalence of non-communicable diseases (NCDs) such as cardiovascular diseases (CVDs) and diabetes constitute a pertinent issue of global healthcare concern [3]. It is projected that NCDs will be the leading cause of disabilities in every part of the world by 2030 [14]. Emerging evidence indicates that low and middle-income countries (LMICs) suffer a higher burden of these diseases [14] as they accounted for 78% of the global deaths and 85% of premature deaths due to NCDs in 2016 [4]. For instance, out of the total 617.3 deaths (due to NCDs) recorded in Nigeria in 2016, the estimated combined deaths due to cardiovascular diseases and diabetes was 251.4 [15]. Also, the risk of dying prematurely from NCDs in Nigeria is estimated at 22% [4]. Similarly, the economic impact of NCDs on families and societies in LMICs can not be overemphasized, especially because families who are already living below the poverty margins suffer the loss of income and are thrust further into extreme poverty due to premature deaths or disabilities from CVDs and diabetes [16, 17].

Medicines are extremely crucial for the primary and secondary prevention, and management of CVDs and diabetes, and if utilized appropriately, drugs can effectively reduce up to 80% of the global burden of NCDs [3]. However, medicines for NCDs remain widely inaccessible and astronomically priced in low-income countries than in high-income countries [17]. For example, in an analysis of 49 medicines for NCDs, only 23.8% of the lowest-priced generics (LPGs) met the World Health Organization target availability in LMICs in comparison to 36.0% in the upper-middle-income countries [18]. In a similar vein, the overall availability of LPG cardiovascular drugs in 36 LMICs was 26.3% in the public sector and 57.3% in the private sector [19]. These disparities in the pricing and availability of medicines are worrisome as a country’s income determines whether its patients will receive at least one medicine for secondary prevention of cardiovascular diseases. In this regard, the probability was 19·8% in low-income countries, 30·7% in low and middle-income countries, and 54·9% for upper-middle-income countries [17].

The World Health Organization/Health Action International (WHO/HAI) developed a standardized method for measuring the price, availability, and affordability of medicines, which facilitates comparison of the results across countries [20]. This method recommends the inclusion of 14 global medicines that represent medicines from different therapeutic groups that are prescribed in most regions of the world. Data on the global drug enable comparison with the therapeutic group being studied and provides data on the access to medicines globally. All the 14 global medicines are included in the World Health Organization (WHO) essential medicine list (EML), but not in the Nigerian EML. Previous studies [19, 21–23] have utilized the WHO/HAI method to investigate the availability and affordability of medicines for different baskets of medicines and reported variability in the availability and affordability of drugs. For example, Mourik *et al*. [19] reported LPG atenolol as the most available cardiovascular medicine in public and private sectors across 36 countries, whereas Captopril and hydrochlorothiazide had the lowest and highest median price ratio (MPR), respectively. The affordability for LPG was 2 days’ wages, whereas Originator Brand (OB) required 8 days’ wages for one month supply of one cardiovascular drug [19]. The sole existing study [22] that has examined the access to essential medicine in Nigeria using the first edition of the WHO/HAI method reported mean availability ranging from 2.4% in the public health sector to 34.1% in private pharmacies. Other studies conducted in Nigeria [24, 25] have evaluated the availability of medicines in health facilities. For instance, in research covering selected states in Nigeria only 18% of the respondents indicated that their drugs were usually available in the public hospitals [23]. Chuku *et al*. [24] investigated the availability of essential medicines in Calabar and reported 44.76% availability in public facilities and 82.3% in private facilities. However, unlike the present study, these studies focused specifically on medicines for communicable diseases. The current reality is that chronic diseases are increasingly becoming the leading cause of global mortality, this necessitates a paradigm shift in health systems from acute to chronic disease management. Also, the WHO/HAI document recommends intermittent monitoring of the availability and pricing of medicines. This is particularly important as Nigeria is considering the implementation of the Universal Health Scheme. Hence, as the Federal Capital Territory (FCT) of the country, Abuja is the perfect starting point for an investigation into the availability, pricing, and affordability of medicines for NCDs [25]. Based on the foregoing, this study sought to ascertain the pricing, availability, and affordability of cardiovascular, diabetes, and global drugs in Abuja, Nigeria.

## Methods

### Study design and setting

A cross-sectional survey was conducted to determine the availability, prices, and affordability of 49 CVDs, diabetes, and global medicines in Abuja (FCT), Nigeria using the WHO/HAI standardized method from 7th February 2019 to 2nd April 2019. Abuja has three tertiary health institutions administered by the Federal Government and thirteen secondary hospitals supervised by the Federal Capital Development Agency (F.C.D.A). Primary healthcare centers are managed by the local government. The tertiary hospitals procure their medicines individually, while the Government Procurement Agency (GPA) handles the procurement of medicines for the secondary hospitals. Private pharmacies and private hospitals independently procure their drugs from wholesalers or through medical/sales representatives. In Nigeria, medicines are dispensed by both the public and private healthcare sectors. The public healthcare system is divided into three tiers, namely the primary, secondary, and tertiary healthcare systems, while the private sector consists of private pharmacies, private hospitals, patent medicine stores, and maternities. The Essential Medicine List (EML) of the Federal Ministry of Health stipulates the type of medicines to be dispensed at each level of healthcare. Presently, CVDs, diabetes, and prescription medicines are primarily stocked at the secondary and tertiary healthcare levels. Private hospitals stock medicines according to their different capacities, while private pharmacies, which are often operated by licensed pharmacists’ stock all classes of drugs. These dynamics make Abuja the perfect case study for the investigation of the availability, pricing, and affordability of medicines for CVDs and diabetes in Nigeria.

### Sampling

The study was conducted in the six administrative areas (Abaji Area Council, Abuja Municipal Area Council (AMAC), Bwari Area Council, Gwagwalada Area Council, Kuje Area Council, and Kwali Area Council) of Abuja (also referred to as the FCT). The sampling method put forward by WHO/HAI informed selection of the facilities [20]. The list of secondary hospitals in Abuja was obtained from the FCT health service department, and an exhaustive list of the tertiary and secondary public hospitals in Abuja comprising 17 public health facilities and 1 Government Procurement Agency (GPA) was compiled. We excluded primary health centers because the drugs under study are prescription drugs that are strictly stocked by secondary and tertiary health facilities. The sampling of the medicine outlets was based on the availability of health facilities with AMAC, Bwari, and Gwagwalada area councils having more health facilities, but only AMAC had more than five public health facilities. The main public health facility in each administrative area was sampled and additional health facilities were randomly chosen. A total of 13 public health facilities (1 in Abaji, 5 in AMAC, 2 in Bwari, 2 in Gwagwalada, 2 in Kuje, and 1 in Kwali), and the GPA were sampled. Private pharmacies and Private Hospital Pharmacies were sampled from the lists obtained from the Association of Community Pharmacists and Association of Private Hospital Owners respectively. Forty private pharmacies and 34 private hospitals were sampled using systematic random sampling from the lists of 59 private pharmacies and 42 private hospital pharmacies respectively. The list of private pharmacies was garnered from the association’s monthly meeting attendance list as the executives refused to furnish us with the complete list of registered private pharmacies in the FCT, although an introductory letter was presented to the researchers. At least five private pharmacies and private hospitals were selected in area councils that have up to five private facilities. The sole private pharmacy in Abaji and the only two private pharmacies in the Kwali Area councils were included. Some private facilities were included as backup outlets when up to 50% of the survey medicines were unavailable in a selected medicine outlet according to the WHO/HAI method. Of the 88 medicine outlets selected, 66 facilities (including 13 public health facilities, 27 private pharmacies, and 25 private hospitals) and one government procurement agency were surveyed. Excluded pharmacies were those that withheld information about the prices of their drugs, private hospital pharmacies that could not demarcate the prices of the drugs from the consultation costs as they were merged into a single bill, and those that did not stock drugs.

### Selection of medicines for the survey

Forty-nine medicines were used in the survey (35 supplementary and 14 global core drugs). Twenty-five (25) cardiovascular drugs and ten (10) anti-diabetics constituted the supplementary list. The researchers’ choice of supplementary medicines was informed by the drug use study that identified the most prescribed CVDs and anti-diabetic drugs in Abuja, and those that constituted up to 3% of the 1008 prescriptions were selected [13]. Atorvastatin was added because simvastatin, which appears on the global list, was seldom used by healthcare providers in Abuja. All the global medicines were included because they are instrumental for the comparison of data on access to medicines globally. This selection is congruent with the WHO/HAI protocol guide for the selection of drugs for a survey [20]. The choice of drugs was intended to encompass the different pharmacological classes of therapeutic agents. The cardiovascular drugs included calcium channel blockers, diuretics, angiotensin-converting enzyme inhibitors, angiotensin receptor blockers, beta-blockers, centrally acting drugs, and cardiac drugs. The anti-diabetic drugs included sulphonylureas, biguanides, dipeptidyl dipeptidase 4 inhibitors, and insulin. Diverse types of insulin were added to ensure the acquisition of detailed data on the availability of insulin.

The WHO/HAI method strongly recommends the collection of data on prices and availability of medicines on specific strength and dosage forms. However, this recommendation was not strictly adhered to in the case of insulin as diverse available strengths of insulin listed in the survey drugs were documented. By doing so, detailed data on the availability of the primary medicines used for cardiovascular diseases and diabetes were obtained.

### Data collection, entry, and analysis

Data were collected by the principal investigator and two trained pharmacists using the medicine price data collection form. Data on the availability of drugs and the prices paid by patients were collected from private pharmacies, public and private hospital pharmacies, respectively. Medicine prices were collected from either the price tags on the drugs or from pharmacy personnel. The data were collected on both the originator brands (OBs) products, and the lowest-priced generics (LPGs) products found on each of the facilities on a specific day. Procurement prices were collected from the procurement offices of two tertiary hospitals and the government procurement agency. The data were then entered into Microsoft excel workbook part 1 designed by WHO/HAI [26]. To ensure data quality, the data collection forms were reviewed daily to eradicate any irregularities in the data collected, and the data on the field data consolidation pages were also manually inspected. Availability, median price ratio (MPR), and affordability were analyzed for each sector. Survey drugs that were not found in the National Agency for Food and Drug Administration and Control (NAFDAC) repository and any medicine outlets were excluded from the analysis to establish accurate availability [20]. The excluded drugs include OBs acetylsalicylic acid 75mg, amitriptyline 25mg, carvedilol 6.25mg, captopril 25mg, digoxin 0.25g, hydrochlorothiazide 25mg, ramipril + hydrochlorothiazide 5mg + 12.5mg, simvastatin 25mg, LPGs insulin glargine and vildagliptin + metformin 50mg + 1000mg. Also, excluded from the analysis were the OB of paracetamol suspension and cotrimoxazole suspension because the manufacturer produced them in licensed indigenous companies under a different name that was included in the generic.

### Calculation of availability, price, and affordability

Availability refers to the percentage of the sampled facilities that have the selected medicines available on the specific day of data collection [20]. The drug must not be expired, it must be fit for the patient’s consumption and must be readily available for collection. Availability was calculated across the sectors for all the survey drugs, cardiovascular, anti-diabetic, and global drugs. Percentage availability was defined as extremely low at 30% or remarkably high at 80% [27]. Medicine prices were expressed as Median Price Ratio (MPR), which is the median unit price in local currency divided by the International Reference Price (IRP) in the local currency. The MPR indicates the discrepancies between the prices of a drug in comparison to the IRP. The IRP are median prices of medicines offered by suppliers to developing and middle-income and was obtained from the 2016 edition of the Drug Price Indicator Guide published by Management Sciences for Health (MSH) [28]. It was calculated in the local currency: the naira, by converting the price of the US dollar to naira on the first day of data collection (at 1$ = N359.68K). The MPR for all the survey medicines included only medicines that had documented prices from up to 4 outlets [20]. To determine the MPR for individual drugs, the target was reduced to one drug to get comparable information for drugs that were not obtainable in at least 4 medicine outlets. The median price ratios were analyzed for medicines with IRP, while others were represented as Median Price (MP). Reasonable public procurement MPR was targeted at ≤1 while the patients’ payment was not expected to exceed 2.5 MPR [27]. Affordability in this context refers to the number of days’ wages required by the lowest-paid unskilled government worker to purchase a one-month supply of cardiovascular drugs, anti-diabetics, and a full course of treatment for acute illness [20]. The cost of treatment required less than a day’s wage for a month’s supply of medicines for cardiovascular diseases and diabetes or a full course of treatment for acute diseases, was considered affordable. Affordability was calculated for LPGs found in at least two survey sectors. The Nigeria treatment guideline was used to estimate the number of tablets needed for supplementary drugs [29]. The Federal Government of Nigeria’s minimum wage of N18, 000 (at the time of the survey) was used to determine the daily pay of the lowest-paid government worker. The affordability was estimated by multiplying the Median Price in local currency by defined daily dose and dividing that by the daily wage of the lowest-paid unskilled government worker.

### Ethics statement

Ethical approval for this study was obtained from the Federal Capital Territory Health Research Ethics Committee Abuja, the National Hospital Abuja, and the University of Abuja Teaching Hospital Gwagwalada. An approval letter was obtained from the district hospitals, and verbal consent was acquired from the management of the private facilities before data collection.

## Results

### Availability of medicines

**Fig 1:**
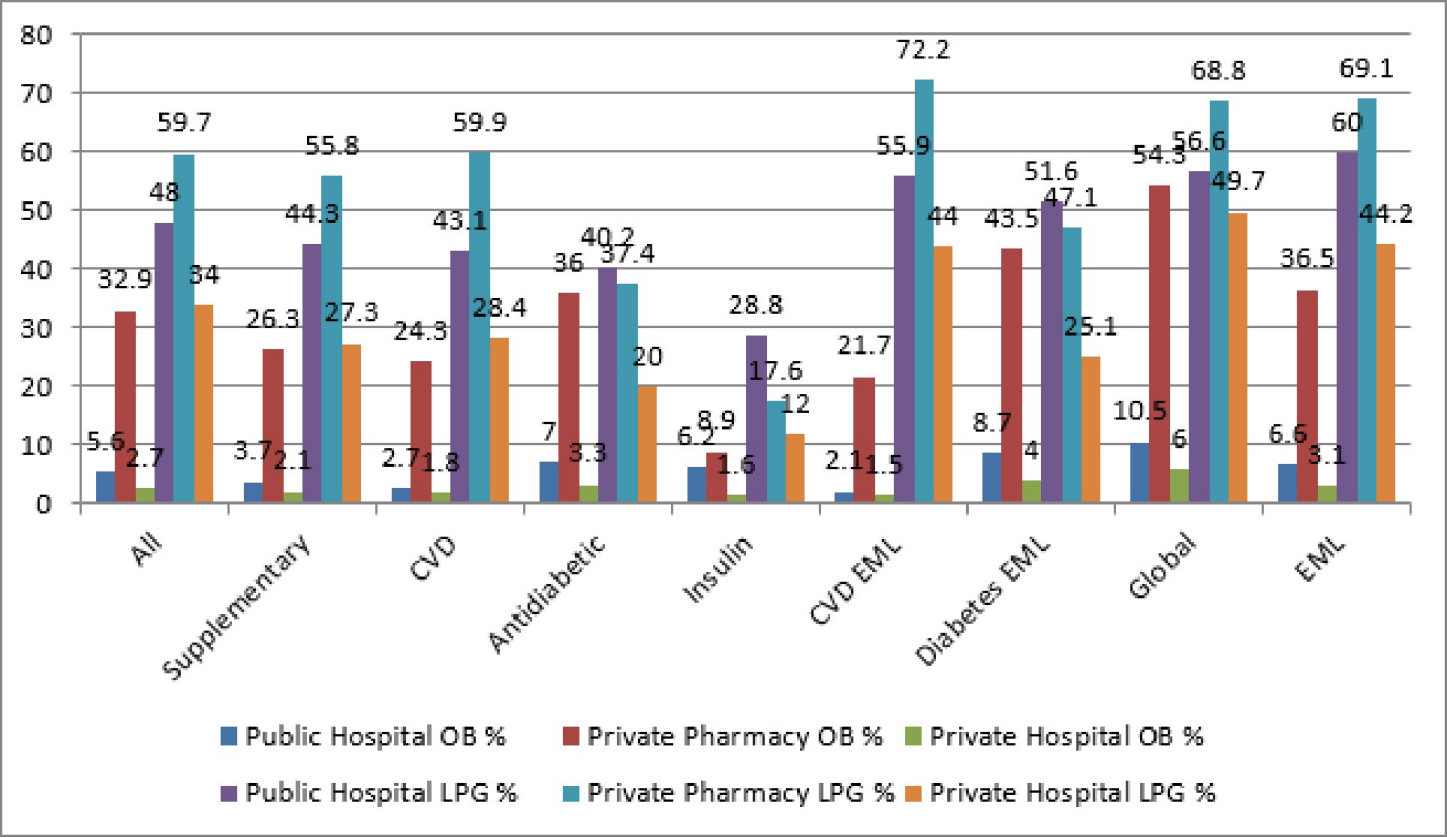
Availability of originator brands and generic medicines across Abuja pharmacies. CVD = Cardiovascular drugs, EML = Essential Medicine List.

The availability of the surveyed drugs is detailed in Fig 1. The mean availability differed in the three types of pharmacies surveyed. The overall mean availability of the OB drugs was 32.9%, 5.6%, 2.8%, while LPGs were 59.7%, 48.0%, 34% in the private pharmacies, public hospitals, and private hospitals pharmacies, respectively. The availability of cardiovascular drugs was highest in the private pharmacies at 24.3% for OBs, and 59.9% for LPGs. Analysis of LPG cardiovascular and anti-diabetic drugs on EML had a higher availability across the three pharmaceutical sectors but were not up to 80% of WHO target availability. Thirty-seven medicines on the Nigeria EML had a mean availability of 69.1% in private pharmacies, 60% in public hospital pharmacies, and 44.2% in private hospital pharmacies.

### Availability of cardiovascular, diabetes, and global drugs

As depicted in **Table 1**, the LPGs were more available as 15, 10, and 6 generic drugs met the WHO target of 80% availability in private pharmacies, public hospitals, and private hospitals respectively. Two generic cardiovascular and four global drugs had greater than 80% availability in the three sectors. In terms of originator brands, only private pharmacies recorded availability of greater than 80% for five medicines, metformin 500mg, diclofenac 50mg, amoxicillin 500mg capsule, ceftriaxone injection, and salbutamol inhaler and none in both the public and private hospital pharmacies. Atenolol 50mg, losartan 50mg, nifedipine sr 20mg OBs were not available across the three types of pharmacies surveyed. Considering the insulin, Biphasic isophane insulin was the most available insulin in Abuja pharmacies.

**Table 1:** Availability of cardiovascular, anti-diabetic, and global drugs by pharmacies.

Drugs	Strength	Public hospital		Private pharmacy		Private hospital	
		OB%	LPG%	OB%	LPG%	OB%	LPG%
**Cardiovascular drugs**							
Captopril	25mg	0.0	0.0	0.0	3.7	0.0	0.0
Lisinopril	5mg	0.0	76.9	3.7	96.3	0.0	36
Lisinopril	10mg	0.0	92.3	3.7	92.6	0.0	60
Irbesartan	150mg	7.7	0.0	33.3	3.7	4	0
Losartan	50mg	0.0	46.2	0.0	70.4	0.0	20
Telmisartan	40mg	0.0	30.8	14.8	48.1	0.0	8
Telmisartan	80mg	0.0	38.5	33.3	37	0.0	4
Valsartan	160mg	7.7	0.0	44.4	7.4	4	0.0
Acetyl Salicylic acid	75mg	0.0	84.6	0.0	100	0.0	88
Clopidogrel	75mg	0.0	30.8	0.0	56.6	0.0	8
Atenolol	50mg	0.0	62.9	0	74.1	0.0	32
Atenolol	100mg	0.0	53.8	3.7	66	0.0	28
Bisoprolol	5mg	7.7	7.7	25.9	40.7	0.0	0
Carvedilol	6.25mg	0.0	7.7	0.0	37	0.0	4
Digoxin	0.125mg	0.0	7.7	0.0	59.3	0.0	24
Methyldopa	250mg	0.0	69.2	33.3	81.5	0.0	72
Amlodipine	5mg	7.7	92.3	59.3	100	4	56
Amlodipine	10mg	7.7	100	77.8	96.3	4	88
Nifedipine SR	20mg	0.0	100	0.0	88.9	0.0	52
Nifedipine XL	30mg	0.0	38.5	3.7	63	0.0	16
Amiloride + Hydrochlorothiazide	5mg + 25mg	0.0	84.6	25.9	100	0.0	72
Furosemide	40mg	0.0	76.9	11.1	74.1	4	40
Hydrochlorothiazide	25mg	0.0	38.5	0.0	66.7	0.0	36
Spironolactone	25mg	0.0	23.1	37	59.3	8	24
Atorvastatin	20mg	0.0	15.4	37	48.1	8	8
Simvastatin	20mg	0.0	0.0	0.0	37	0.0	4
Amlodipine + Valsartan	5mg +160mg	15.4	7.7	37	29.6	4	0.0
Ramipril + Hydrochlorothiazide	5 + 12.5mg	0.0	15.4	0.0	40.7	0.0	8
**Anti-diabetic drugs**							
Metformin	500mg	15.4	100	92.6	92.6	8	68
Metformin	1000mg	15.4	38.5	70.4	33.3	0	8
Glibenclamide	5mg	0	46.2	74.1	77.8	0	60
Gliclazide	60mg	0	0	14.8	0	4	0
Glimepiride	4mg	7.7	61.5	51.9	63	4	0
Biphasic Isophane Insulin	100units/ml	15.4	69.2	25.9	37	4	20
Insulin glargine	100units/ml	15.4	0	18.5	0	4	0
insulin isophane	100units/ml	0	0	0	7.4	0	4
insulin zinc	100units/ml	0	0	0	0	0	0
Soluble insulin	100units/ml	0	46.2	0	25.9	0	24
Vildagliptin + Metformin	50mg + 1000mg	7.7	0	48.1	0	4	0
**Global drugs**							
Amitriptyline	25mg	-	76.9	-	74.1	-	32
Amoxicillin	500mg	0.0	92.3	88.9	92.6	4	80
Ceftriaxone injection	1g	30.8	100	85.2	92.6	16	80
Ciprofloxacin	500mg	0	92.3	14.8	96.3	0	76
Co-trimoxazole suspension	8 + 40 mg/ml	0	23.1	0	88.9	0	56
Diazepam	5mg	0	61.5	0	51.9	0	52
Diclofenac	50mg	7.7	53.8	92.6	96.3	0	88
Omeprazole	20mg	0	69.2	0	96.3	-	64
Salbutamol inhaler	200mcg	53.8	15.4	88.9	7.4	20	0
Paracetamol suspension	24mg/5ml	0	100	0	92.6	0	96
Simvastatin	20mg	0	0	-	37	0	4

OB = Originator Brand, LPG = Lowest Priced Generic, PHP = Public Hospital Pharmacy, PP = Private Pharmacy, PRHP = Private Hospital Pharmacy.

### Medicine prices

#### Procurement prices

**Fig 2:**
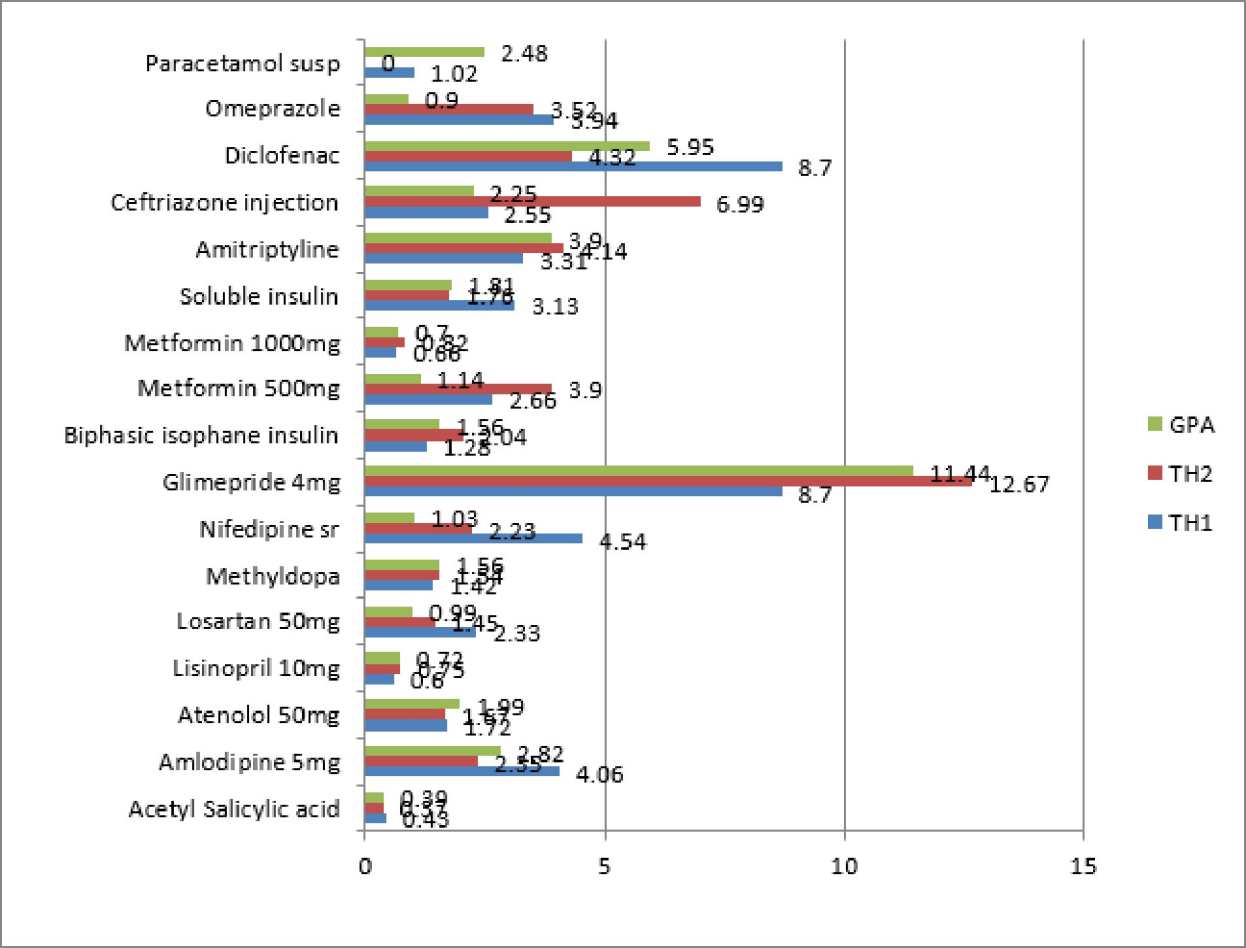
Procurement prices for generic medicines in Abuja. GPA = Government procurement Agency, TH1 = Tertiary Hospital 1, TH2 = Tertiary Hospital 2.

The procurement MPR of all the medicine groups is presented in **Table 2**. The overall procurement MPR for 9 OBs was 9.60, whereas 33 LPGs were procured at 1.72 times the International Reference Price. Procurement prices for the survey medicines ranged from (0.39–11.14) LPGs and (1.97–37.59) OBs. Fig 2 shows MPR for generic medicines found in at least two procurement offices. Generally, three LPGs acetylsalicylic acid, lisinopril 10mg, and metformin 1000mg were procured at less than 1MPR by the three procurement agencies. Several drugs showed considerable variation in their procurement prices.

**Table 2:** Procurement prices for originator brands and lowest priced generics medicine.

Medicine list	OB/LPG	n	MPR	25%tile	75%tile	Minimum	Maximum
All	OB	9	9.60	1.75	23.92	1.52	37.59
	LPG	33	1.72	1.12	2.66	0.39	11.14
Supplementary	OB	4	9.76	7.69	14.38	1.97	27.72
	LPG	22	1.55	1.12	2.56	0.39	11.44
CVDs	OB	3	9.60	5.75	9.76	1.91	9.93
	LPG	18	1.45	1.10	2.18	0.39	5.37
CVD EML	OB	2	9.76	9.68	9.85	9.60	9.93
	LPG	15	1.54	1.23	2.47	0.39	5.37
Diabetes	OB	3	9.42	5.72	18.59	1.97	27.72
	LPG	6	2.24	1.62	3.52	0.70	11.44
Diabetes EML	OB	3	9.42	5.72	18.59	1.97	27.72
	LPG	6	2.24	1.62	3.52	0.70	11.44
Global	OB	5	9.46	1.91	23.92	1.52	37.59
	LPG	11	2.39	1.40	3.09	0.56	5.95
EML	OB	7	9.46	1.94	25.82	1.52	37.59
	LPG	27	1.81	1.37	3.09	0.39	11.14

CVDs = Cardiovascular diseases, EML = Essential Medicine List, OB = Originator Brand, LPG = Lowest Priced Generic, n = number of medicines.

#### Patient prices

The median price ratio of the individual and medicine lists is summarized in **Table 3**. The patient generic MPR ranged from 1.89 in the private pharmacies to 10.35 in the private hospitals. Public hospitals and private pharmacies showed reasonable and comparable generic MPRs. A few LPGs had low MPRs across the three sectors. Four and five generic medicines had < 1 MPR, in the public hospitals and the private pharmacies respectively, which indicates that Nigerians pay less than the IRP for these drugs. Also, four, 19, and 20 generic medicines showed ≤ 2.5MPR in the private hospitals, public hospitals, and private pharmacies respectively. Two OBs, bisoprolol 5mg and salbutamol inhaler were sold at less than 2.5 times their IPR in public hospitals and private pharmacies. The OBs were sold higher than LPGs, with OB furosemide having the highest MPR at 189 in private hospitals. Generic medicines with greater than 30 (MPR) were seen only in private hospitals.

**Table 3:** Patient median price ratio (MPR) for medicine list and individual drugs.

	ORIGINATOR BRAND			LOWEST PRICED GENERIC		
Medicines lists	PHP	PP	PRHP	PHP	PP	PRHP
	MEDIAN PRICE RATIO					
All surveyed medicines	13.56	8.08	16.38	2.66	2.60	7.89
Supplementary	0	6.47	0	2.14	2.47	7.89
Cardiovascular	0	5.98	0	2.07	1.89	7.65
Diabetes	0	11.03	0	2.39	2.35	8.95
Insulin	0	2.44	0	2.09	2.29	4.85
Global	13.56	11.03	16.38	3.49	2.90	10.35
Essential Medicine List	13.56	12.83	16.38	2.67	2.94	7.89
Individual Medicines							
Acetyl Salicylic acid 75mg	NA	NA	NA	1.0	0.6	5.95
Amlodipine 5mg	6.2	21.1	38.7	3.5	3.8	8.13
Amlodipine 10mg	9.7	15.0	36.4	2.3	1.6	5.17
Atenolol 50mg	NA	NA	NA	2.8	4.6	11.6
Atenolol 100mg	NA	29.3	NA	1.7	4.2	8.4
Atorvastatin 20mg	NA	6.5	10.9	1.3	0.9	3.1
Bisoprolol 5mg	2.3	2.1	NA	0.9	0.8	NA
Captopril 25mg	0	0	0	0	2.4	0
Carvedilol 6.25mg	NA	NA	NA	2.0	1.6	5.0
Clopidogrel 75mg	NA	NA	NA	0.7	0.9	1.8
Digoxin 0.125mg	0	0	0	6.9	6.9	22.0
Furosemide 40mg	0	36.1	189.9	5.0	6.5	22.8
Hydrochlorothiazide 25mg	NA	NA	NA	6.5	6.5	32.3
Lisinopril 5mg	0	3.2	0	1.9	1.6	3.9
Lisinopril 10mg	0	2.7	0	0.7	0.7	1.8
Losartan 50mg	0	0	0	1.2	1.4	2.0
Methyldopa 250mg	0	4.7	0	1.7	2.2	4.0
Nifedipine SR 20mg	0	0	0	2.7	3.0	8.2
Nifedipine XL 30mg	0	11	0	4.1	6.1	11.6
Simvastatin 20mg	NA	NA	NA	0	1.6	2.7
Spironolactone 25mg	0	5.5	9.7	2.4	2.6	7.7
Biphasic Isophane Insulin 100units/ml	2.2	2.4	5.82	2.0	2.5	14.54
Glibenclamide 5mg	0	14.6	0	6.6	6.3	31.7
Gliclazide 60mg	0	8.08	0	0	0	0
Glimepiride 4mg	36.8	47.5	64.2	16	19	0
Insulin isophane 100units/ml	0	0	0	0	1.8	2.3
insulin zinc 100units/ml	0	0	0	0	0	0
Metformin 500mg	12.1	11	23.8	2.7	2.2	13.2
Metformin 1000mg	2.5	2.2	0	1.1	1.2	2.8
Soluble insulin 100units/ml	0	0	0	2.1	2.1	3.9
Amitriptyline tablet 25mg	NA	NA	NA	5.0	3.3	18.8
Amoxicillin capsule 500mg	0	5.6	12.1	1.9	1.6	4.6
Ceftriaxone injection 1g	25.2	24.5	29.0	3.5	3.5	6.7
Ciprofloxacin tablet 500mg	0	61.7	0	3.7	3.0	7.5
Co-trimoxazole suspension 8+40mg/ml	NA	NA	NA	2.3	2.9	4.8
Diazepam tablet 5mg	0	0	0	0.6	2.9	14.5
Diclofenac tablet 50mg	49.4	49.4	0	12.4	6.2	30.9
Omeprazole tablet 20mg	0	0	0	7.9	4.2	14.1
Salbutamol inhaler 200mcg	2.0	2.3	3.8	1.9	1.5	0
Paracetamol suspension 24mg/5ml	NA	NA	NA	2.7	1.8	4.5

n = number of medicines included in the analysis, PHP = Public Hospital Pharmacy, PP = Private Pharmacy, PRHP = Private Hospital Pharmacy, NA = Not available.

#### Comparing median price ratios across sectors

**Table 4** shows the percentage price differences between LPG procurement and patient prices in the sectors. Patient prices were higher than the procurement prices. The percentage price difference between the procurement prices and patient prices for LPGs was 51.4% in private pharmacies, 49.4% in public hospitals, and 323% in private hospital pharmacies. Patient prices were 62.8% lower for 28 LPGs in private pharmacies compared to private hospitals. A direct comparison of the price differences between OB products was not possible due to unavailability in the public and private hospital pharmacies.

**Table 4:** The percentage price difference between lowest priced generics procurement and patient prices.

Sector	N	% Price difference
Procurement–public	28	49.4
Procurement–Private Pharmacy	33	51.4
Procurement–Private hospital Pharmacy	27	32
Private hospital–private pharmacy	28	-62.8
Private hospital–public hospital	25	204.2
Public hospital–private pharmacy	28	10.5

n—number of drugs.

#### Affordability of medicines

The affordability of survey drugs whose LPGs were found in at least two sectors is summarized in **Table 5**. No originator brand was affordable across the three pharmacies surveyed. Treatments that cost less than a day’s wage included: acetylsalicylic acid 0.2, hydrochlorothiazide 0.5 in the private pharmacies, and acetylsalicylic acid 0.3, atenolol 50mg 0.9, and hydrochlorothiazide 25mg tab 0.5 in the public hospitals. Additionally, 3, 6, and 10 global medicines were affordable in private hospitals, public hospitals, and private pharmacies respectively. Treatment with insulin required 6–17 days wage, while nifedipine and atorvastatin required 2–6 days wage.

**Table 5:** Affordability of cardiovascular, anti-diabetic and global drugs.

Drug name	STRENGHT	LOWEST PRICED GENERIC			ORIGINATOR BRAND			
Cardiovascular		PHP	PP	PRHP	PHP	PP	PRHP	Dose
Acetylsalicylic acid	75mg	0.3	0.2	1.6	-	-	-	1tabx30
Amlodipine	5mg	2.3	2.5	5.4	4.1	14.1	25.8	1tabx30
Atenolol	50mg	0.9	1.5	3.7	-	-	-	1tabx30
Atorvastatin	20mg	-	1.7	6.1	-	12.5	21.1	1tabx30
Bisoprolol	20mg	3	2.5	-	7	7.5	-	2tabx30
Digoxin	0.25mg	1.3	1.3	4	-	-	-	1tabx30
Hydrochlorothiazide	25mg	0.5	0.5	2.5	-	-	-	1tabx30
Lisinopril	5mg	3	2.6	6.3	-	-	-	1tabx30
Losartan	50mg	2.5	2.9	-	-	-	-	1tabx30
Methyldopa	250mg	3	3.8	6.9	-	8.3	-	3tabx30
Nifedipine SR	20mg	2	2.2	6	-	-	-	2tabx30
Simvastatin	20mg	-	1.5	2.5	-	-	-	1tabx30
Biphasic isophane	100units/ml	5.8	7.1	16.7	6.3	7	41.7	10mls
Glibenclamide	5mg	0.7	0.7	3.3	-	1.5	-	30tabs
Gliclazide	80mg	-	-	-	-	7.1	-	30tabs
Glimepiride	4mg	3.4	4	-	7.8	10	13.5	30tabs
Metformin	500mg	2.1	1.8	10.7	9.8	8.9	19.3	90tabs
Soluble insulin	100units/ml	7	7.5	13.8	-	-	-	10ml
Amitriptyline	25mg	2.3	1.5	8.5	-	-	-	90tabs
Amoxicillin	500mg	0.7	0.5	1.8	-	2.1	4.6	21tabs
Captopril	25mg	-	2.1	-	-	-	-	2tabx30
Ceftriaxone injection	1g	0.8	0.8	1.6	6.0	5.8	6.8	1vial
Ciprofloxacin	500mg	1.2	0.9	2.3	-	19.4	-	14tabs
Co-trimoxazole suspension	8 + 40 mg/ml	0.5	0.6	1	-	-	-	70mls
Diazepam	5mg	0.0	0.1	0.6	-	-	-	7tabs
Diclofenac	50mg	2	1	5	8	8	-	60tabs
Omeprazole	20mg	2	1.1	3.6	-	-	-	30tabs
Salbutamol inhaler	200mcg	2.1	1.7	-	2.2	2.5	4.2	200dose
Paracetamol suspension	24mg/5ml	0.4	0.2	0.6	NA	NA	NA	45mls

OB = Originator Brand, LPG = Lowest Priced Generic, PHP = Public Hospital Pharmacy, PP = Private Pharmacy, PRHP = Private Hospital Pharmacy.

## Discussion

This study surveyed the availability, prices, and affordability of cardiovascular, anti-diabetic, and 14 global medicines in Abuja, Nigeria. The motivation for this study was instigated by the dearth of recent data on the availability and variability in prices and affordability of cardiovascular and diabetic medicines in Abuja. As far we are aware, until the current date, no study has compared the prices and availability of cardiovascular and anti-diabetic drug alternatives across three different healthcare sectors of a single state in Nigeria. This paper is not only relevant, but it also presents an overarching insight into the availability, pricing, and affordability of cardiovascular, anti-diabetic, and global medicines in Nigeria using the Federal Capital Territory (Abuja) as a microcosm. The findings in this paper are timely and would prove invaluable to policymakers, especially because Nigeria is currently considering Universal Health Coverage. Thus, the information herein on the availability, pricing, and affordability of these medicines will be indispensable to the formulation and implementation of policies that will ensure effective access to medicines in Nigeria.

### Availability of medicines

The availability of surveyed medicines across all the sectors was lower than the 80% recommended by the WHO Global Action Plan for Prevention of NCDs. This finding is consistent with previous results from other low and middle-income countries [30, 31]. This could be because our survey list consisted mainly of prescription drugs which have also shown lower availability when compared to over the counter drugs in high-income countries [21]. It is not surprising that the availability was even lower for OB products. This is possibly because they are unreasonably overpriced and consequently seldom prescribed or in demand. It is also possible that patients opt for the more affordable generic versions of medicines even when the originator brands are prescribed. Similarly, the list of medicines on the Nigeria EML had low availability even in public hospitals. This might imply that the EML does not necessarily influence the prescription and procurement processes in public hospitals. However, CVDs and anti-diabetic drugs on the Nigeria EML [32] were more available, which is consistent with [33]. This could be because they are in high demand by prescribers and are widely accepted. Likewise, the high availability of the basket of drugs on the global list is attributable to the fact that the medicines belong to different classes.

The high availability of LPG calcium channel blockers (amlodipine and nifedipine) and ACEI lisinopril in our study differs from previous reports of atenolol as the most available cardiovascular drug in LMIC [19], and enalapril [31] in Sri Lanka. This high availability of the aforementioned drugs in our study can be linked to the preferred recommendation of CCBs to black patients [34] and the preference of ACEI lisinopril by the prescribers. Also, the fact that our results were generated from one country might indicate conformity to a specific treatment pattern. In contrast to a previous study [19] in low and medium-income countries, the OBs atenolol, captopril, and nifedipine sr were not available in the surveyed pharmacies in our study. This may be due to the prevalent availability of affordable generic brands. The global cardiovascular medicines, bisoprolol, and captopril had lower availability than a recent report in Malawi [35]. Bisoprolol’s low availability can be attributed to its newness in the Nigerian market and non-inclusion in the EML. It then implies that most patients who require a beta-blocker will have to depend on the more available atenolol as carvedilol had low availability. Although captopril is on the Nigeria EML, it was less available than bisoprolol, which is newer and not yet included on the Nigeria EML. Availability of captopril plummeted from 21.4% availability in Nigerian public hospitals in 2006 [22] to 0%. This is in stark contrast to the recent report of 100% availability of captopril in Sri Lankan public hospitals [31]. The decline of captopril’s availability is likely due to the preference of lisinopril by prescribers in the FCT [13]. Insulin generally had low availability. Its highest availability was in public hospitals, and biphasic isophane insulin was the most available. This is in agreement with a Sri Lanka study [31]. This indicates that insulin continues to be widely unavailable to the majority of the world’s population [36]. The low availability recorded across all pharmacies is probably because of inadequate financing of the health sector in Nigeria as the government’s health expenditure was 6.5% of the total government expenditure in 2013 [11]. Since health resources are limited, pharmacies would likely stock drugs that retail faster. This means that patients will have to visit more than one pharmacy to acquire a complete supply of their medicines. This poses a further limitation to their access to the much-needed medicines.

However, some LPG cardiovascular medicines had > 80% availability with 8 in the private pharmacies, 5 in the public hospitals, and 2 in the private hospitals, which is higher than previous reports from both Nigeria and Cameroon [23, 37]. This may be linked to our comprehensive list of cardiovascular medicines. The fact that metformin 500mg was more available in our findings than in a China report [38] shows that it is the preferred drug utilized by Nigerians to regulate their blood sugar. The availability of global drugs in public hospitals was better when compared to a report in Malawi [35]. This is possible because availability data depends on one spot measurement rather than on the monthly average. Five OB drugs on the global list used mainly for the treatment of acute diseases (except metformin 500mg) showed > 80% availability in private pharmacies. In comparison to a 2006 report [22], which recorded that only one generic cardiovascular drug was available in more than 80% of Nigeria private pharmacies, and none in both public and private hospitals. Our finding represents an improvement in medicine availability in the Nigerian healthcare sector. This improvement is likely due to the increased prescription of those drugs [13, 39], awareness, increased demand, and acceptance.

Although individual drugs have high availability as a result of differences in the prescription volume of drugs in the same therapeutic or even pharmacological group, access to essential medicines remains perturbingly low. Considering the fact that access to medicines is a human right [17], low or sparse availability is unacceptable. Having only one drug available from an entire pharmacological class (as was the case with the ACEIs, captopril 25mg which had minimal availability of 3.7% in private pharmacies, while lisinopril had almost 100% availability in public hospital and private pharmacies) deprives prescribers and patients of having a wider range of options. The most worrisome finding in this study pertains to the overall sub-optimal availability of insulin across all surveyed sectors (lower than the WHO 80% recommendation).

### Medicine prices

As would be expected, OB products were procured at a higher MPR than the LPGs. The procurement MPR for OBs (9.60) and LPGs (1.72) was higher than the considerable procurement MPR of one [27], and procurement MPR in an Indian government establishment (0.53–0.82) [40], but far lower than that reported in a low-income country, Comoros (the OB was 11.60 and LPG was 3.83) [41]. Procurement prices in Nigeria are probably higher than India due to the limited manufacturing of drugs in Nigeria. In comparison to the Comoros study, our procurement prices were collected from only government establishments and there was a difference in our list of survey drugs. However, acetylsalicylic acid, lisinopril 10mg, and metformin 1000mg were procured at less than 1IRP by the three procurement agencies. A remarkable difference was noted in the procurement prices for some drugs in the three government establishments. This corroborates a report from India [40]. This is probably due to the distinct brands of generic medicines procured by the three procurement agencies. In contrast to earlier findings from 36 countries [19], all procurement MPRs in this study were lower than patients’ prices. The percentage price difference between the procurement MPRs and patients’ prices was higher in private hospitals (324%) than in private pharmacies (54.1%). Based on this finding, one can argue that the populace has a deeper sense of trust in private hospitals than they do for private pharmacies. On the flip side, it could be that the patients are not as knowledgeable about medicines as to easily distinguish between OBs and generics.

Patients paid 13.56 times the IRP for OB products in the FCT’s public hospital pharmacies, as against the reasonable 2.5 MPR [42] and MPR (8.03) recorded for OBs in Sudan public pharmacies [30]. This can be attributed to the available drugs used in the calculation of the overall MPR. For instance, the OB MPR was lower in private pharmacies (8.08) when compared to private retail pharmacies in Sudan (19.37) [30], and Brazil (18.99) [43]. These results suggest that, in comparison to other countries, private pharmacies in Nigeria offer competitive prices for OB products. Patients’ median price ratio for generic medicines in the public hospitals (2.66), and private pharmacies (2.60), is lower compared to Comoros’ public pharmacies (4.45) and private retail pharmacies (5.34) [41]. A possible explanation might be the fierce market competition among the pharmacies which makes price reduction a strategy for attracting and retaining patronage. Patients paid more for LPGs in private hospitals, even higher than the OBs in private pharmacies. Surprisingly, prices were better in the private pharmacies, compared to the government-sponsored public hospitals. Private pharmacies offered patients an alternate opportunity to purchase drugs at cheaper costs, including the drugs that were generally unavailable in public hospitals. The discrepancy in the pricing may be as a result of the different methods of drug procurement employed by the public pharmacies and private pharmacies, specifically concerning the numerous intermediaries involved in the public sector procurement. The high MPR in private hospitals could be linked to a tendency to recover other expenses from drug sales. In our study, the basket of the drugs for EML had the highest OB MPR in the private pharmacies. This differs from the global list in Sudan [30]. This could arguably be due to the differences in the drugs on the Nigeria EML, and the global list. Concerning LPGs, global drugs had the highest MPRs across the pharmacies. This finding is congruent with a recent study conducted in Boston [21]. The high MPR between drugs on the EML and global drugs is plausible because all the global drugs are listed on the Nigeria EML, except two CVD drugs: bisoprolol and simvastatin, which had low availability. It can be argued that medicines on the EML are pricier and more readily available in Nigeria.

The OB furosemide had the highest MPR among the cardiovascular drugs, followed by atenolol, consistent with a previous study in Sudan [30]. Since the OBs of these drugs are mostly unavailable, this might be inconsequential. More importantly, the predominately available LPGs had higher MPRs across all the pharmacies compared to other LPG cardiovascular drugs, possibly due to the high-profit margin. This has a negative implication on the achievement of the WHO global target of access to affordable generic essential medicines for the treatment of cardiovascular diseases in Nigeria. However, OB of bisoprolol and salbutamol inhaler had an MPR of less than 2.5. This indicates that the price of OB can be at the same level as the generic when faced with tougher price competitions in Nigeria. The OB of the anti-diabetic drug, glimepiride, showed the highest MPR, which differed from the findings on glibenclamide in Sudan [30]. Glimepiride is a newer (2nd generation) sulphonylurea that is largely preferred by prescribers in Nigeria [13]. Besides, glimepiride was not included in the Sudan survey. Although the prices were not adjusted, patients paid lower prices for all the drugs in the private pharmacy except for OB ciprofloxacin compared to the 2006 Nigeria study [22]. This is likely due to the increased availability of drugs and competition among pharmaceutical companies.

### Affordability of medicines

Several factors such as the availability, dosage, and pricing of drugs significantly affect their affordability. On average, a thirty-day treatment for cardiovascular, diabetes, and full course treatment for acute illness costs more than a day’s wage in Nigeria. Only three cardiovascular, one anti-diabetic, and five global generic drugs cost less than a day’s wage. The findings of our study differ from those of a study in China [38], in which only three drugs cost more than a day wage. The monthly costs required for the treatment of coronary heart disease in our study amount to at least six days’ wages, which is higher than Brazil’s (5.1 days’ wages), and Bangladesh’s (1.6 days’ wages) [44]. This already exorbitant affordability soars when considering the monthly management of diabetes using a multidrug regimen, for example, purchasing two OB drugs: OB gliclazide + metformin in private pharmacies costs 16 days’ wages as opposed to 5.9 days’ wages in China [38]. These findings notwithstanding, there has been an improvement in affordability in Nigeria since 2006, when the same dosage of glibenclamide required 3.3 days’ wages in public hospitals and private pharmacies, and 4.9 days’ wages in private hospitals [22]. The poor affordability of medicines in Nigeria is attributable to the profit maximization in the private sector due to the unavailability of the drugs, especially the OBs in the public sector. Furthermore, OB anti-diabetic drugs are vastly prescribed across Nigeria [13], which makes the demand higher. From an economic standpoint, most unskilled Nigerians earn less than the N18, 000 monthly federal government minimum wage. Even worse, the majority of Nigerians are unemployed and this complicates the economic and financial burden of long-term treatment of chronic diseases. For example, if a family that is treating a loved one with diabetes using metformin and glibenclamide has to treat an acute infection with ciprofloxacin within a specific month, they would require four days’ wages in a public hospital, 3.6 days’ wages in a private pharmacy, and 16 days’ wages in a private hospital for treatment with LPG. If the OB metformin is prescribed, the treatment will necessitate 11.7 days’ wages in a public hospital, 10.6 days’ wages in a private pharmacy, and 24.9 days’ wages in a private hospital. So, in spite of the seeming affordability of medicines in the private sector, the multitude of low and middle-income earners in Nigeria will still be beleaguered by their costs. This is compounded by the fact that chronic disease treatment requires life-long therapy to pre-empt life-threatening complications and disability.

The standardized WHO/HAI method posed some limitations which we tried to circumvent in our study. For instance, the method evaluates the specific dosage and strength of a particular drug, whilst excluding its alternatives. By focusing on cardiovascular and anti-diabetic drugs, this study strengthened the WHO/HAI method by presenting data on alternate dosages and strengths of the same medicine. Another limitation concerns the determination of availability data based on one visit to the pharmacy. This might not accurately reflect the average availability over time. We argue instead that a sampling of many facilities over a period (preferably three months) would provide better insights into the realistic availability of specific medicines in a particular place. This study does not account for the quality of the medicines studied.

## Conclusion

This study set out to provide invaluable insights into the availability, prices, and affordability of cardiovascular, anti-diabetic, and global drugs in Abuja, Nigeria. The overall availability of originator brands and lowest priced generics was low across the three different sectors that were surveyed, especially for cardiovascular and anti-diabetic drugs. Substantial differences were noted between the prices and affordability of medicines in the different pharmaceutical sectors. Although private pharmacies offered the best availability, prices, and affordability for the surveyed medicines, the prices remained considerably exorbitant in the other sectors. Given the exponential prevalence of NCDs in Nigeria, the Nigerian government must scale up the health budget, initiate policy changes that would facilitate the availability and affordability of medicines, and expedite the implementation of the Universal Health Scheme Program.
